# Survival rate and predictors of mortality among TB/HIV co-infected adult patients: retrospective cohort study

**DOI:** 10.1038/s41598-022-23316-4

**Published:** 2022-11-01

**Authors:** Mesfin Esayas Lelisho, Teramaj Wongel Wotale, Seid Ali Tareke, Bizuwork Derebew Alemu, Sali Suleman Hassen, Daniel Melese Yemane, Birhanu Bedada Korsa, Namso Geda Bedaso

**Affiliations:** 1 Department of Statistics, College of Natural and Computational Science, Mizan-Tepi University, Tepi, Ethiopia; 2 Department of Statistics, College of Natural and Computational Science, Mettu University, Mettu, Ethiopia; 3 Department of Biology, College of Natural and Computational Science, Mizan-Tepi University, Tepi, Ethiopia; 4 Department of Statistics, College of Natural and Computational Science, Madda Walabu University, Bale Robe, Ethiopia

**Keywords:** HIV infections, Tuberculosis, Public health, Statistics

## Abstract

Nowadays, Tuberculosis remains the major cause of HIV-associated mortality, which accounts for 1 out of every 5 HIV-related mortality worldwide. This study aimed to determine the survival rate and predictors of mortality among TB/HIV co-infected patients. An institution-based retrospective cohort study was undertaken on adult TB/HIV co-infected individuals between 1st February 2014 and 30th January 2022 at Mettu Karl Referral Hospital. A Cox regression model was used to identify predictors of survival time to death among TB/HIV co-infected patients. This study comprised 402 TB and HIV co-infected adult patients. Among these, 84 (20.9%) died, and 318 (79.1%) were censored. The study subjects have been followed up for 6920 person-months with an overall median survival time of 17.6 months. The overall incidence rate was 12.1 per 1000 person months [95% CI: 9.77–14.98]. The results of a multivariable Cox regression analysis showed that being at an older age, urban residence, WHO clinical stage II & IV, CD4 count of ≥ 200 cells/mm^3^, bedridden functional status, using INH, and using CPT were associated with the survival time of TB and HIV co-infected patients at a significance level of alpha = 0.05. This retrospective study found that high mortality of TB/HIV co-infected patients occurred in the earlier months of treatment initiation. Close monitoring of patients with low CD4, who do not utilize CPT, who are in advanced WHO stages, and who have poor functional levels can help them improve their health and live longer.

## Introduction

Tuberculosis (TB) is the most frequent opportunistic illness among people living with HIV, and people who are co-infected with TB and HIV are at significant risk of mortality due to the bidirectional link between the two infections^1^. TB has been shown to hasten the progression of HIV infection to AIDS^2^. Persons living with HIV have a 30-fold higher risk of developing TB compared to people who do not have the virus, and the risk of mortality among co-infected persons is nearly twice that of HIV-infected people alone^3^. In 2017, over 0.3 million people died worldwide due to TB and HIV co-infection^4^.

The devastating repercussions of the syndemic interaction between the TB and HIV epidemics have been felt all across the world, with Africans bearing a disproportionate share of the burden^5^. In many countries, particularly in SSA, HIV is fueling the TB epidemic^6^. HIV has also resulted in social, economic, and political catastrophes. Stigma, high vulnerability to additional comorbidities such as mental diseases, the expense of treatment, a rising number of orphans, lower productivity as a result of long-term illness, and the absence of people from the workplace are the most typical outcomes^7,8^. In most cases, co-infection of TB and HIV affects the productive portion of the population, exacerbating the crisis, particularly in low-income nations such as Ethiopia^9^.

According to a report by the world health organization (WHO), Ethiopia is among 20 countries in the world with the highest prevalence of TB and HIV co-infection^10^. In Ethiopia, from 40 up to 70% of HIV patients also have tuberculosis^11,12^. To battle the disease's spread and timeliness, WHO suggests a variety of combined measures for HIV/TB co-infection, such as initiating antiretroviral therapy (ART) to minimize the risk of morbidity and mortality related to HIV, or improving the life quality for HIV-positive peoples^13^. Despite the WHO recommendation that all TB/HIV co-infected patients start ART regardless of their baseline CD4 cell level, Ethiopia's national annual performance report found that ART coverage of patients co-infected with TB and HIV was 70%^14^.

The bidirectional connection between HIV and TB has been observed in the retrospective cohort and case-control studies undertaken in various locations in Ethiopia. For example, a nationwide retrospective cohort study found that 9.0% of HIV patients on treatment were also co-infected with TB^15^. Another study in Ethiopia's Amhara region found that 27.7% of HIV patients on antiretroviral therapy (ART) got tuberculosis (TB)^16^. According to a study conducted in Bahirdar, 30.6% of TB patients were also HIV positive, and one-fifth of TB-HIV cases were deadly^6^. Furthermore, in most studies, TB-HIV-related mortalities were found to be substantially correlated with baseline CD4 cell count, baseline functional level, WHO clinical stage, cotrimoxazole prophylaxis, and co-existence of other OIs among persons co-infected with HIV/TB^6,15,17–20^. Age, gender, occupation, educational status, place of residence, weight, and clinical presentation of TB are also found to be risk factors for death among TB/HIV co-infected patients^21,22^.

Despite the existence of ART in the country, mortality among people who are co-infected with TB and HIV has not decreased^3^. Although, co-infection with TB and HIV is bidirectional and a dual public health burden worldwide, TB/HIV co-infected patients have not received enough attention. Furthermore, the current study location is close to Gambella, one of Ethiopia's regions with the highest rates of TB/HIV co-infection, and a large number of TB/HIV co-infected patients visit Mettu Karl Referral Hospital (MKRH), but no study has been conducted on the survival rate of TB/HIV co-infected patients in the study area. Generally, this study answered the following basic research questions: (i) What are the survival rates of TB/HIV co-infected patients? (ii) What is the overall median survival time and the overall incidence rate per person-month of mortality among TB/HIV co-infected patients? (iii) Which factors are statistically significant predictors of mortality in TB/HIV co-infected patients? Hence based on these questions, the goal of this study was to determine the survival rate and predictors of mortality among TB/HIV co-infected patients in the case of Mettu Karl Referral Hospital. Knowing the predictors impacting survival time is crucial for a country striving to achieve successful TB and HIV control, and to enhance TB/HIV co-management. Furthermore, this could lessen the human suffering and economic burden connected with both diseases, as well as provide recommendations to the relevant authority for the implementation of healthcare infrastructures to reduce diagnostic and clinical care issues in hospitals.

## Material and methods

### Study design and setting

A current study utilized a retrospective cohort study design. The data set was obtained from Mettu Karl Referral Hospital (MKRH), Oromia region. In this health institution, patients were tested under any of the HIV counseling and testing procedures, including voluntary counseling testing (VCT) and provider-initiated HIV counseling and testing units. After HIV is identified in a patient with TB, he/she will be enrolled to start co-trimoxazole preventive therapy (CPT) and, HIV chronic care^23^.

### Sample size and sampling procedure of study subjects

**Figure 1:**
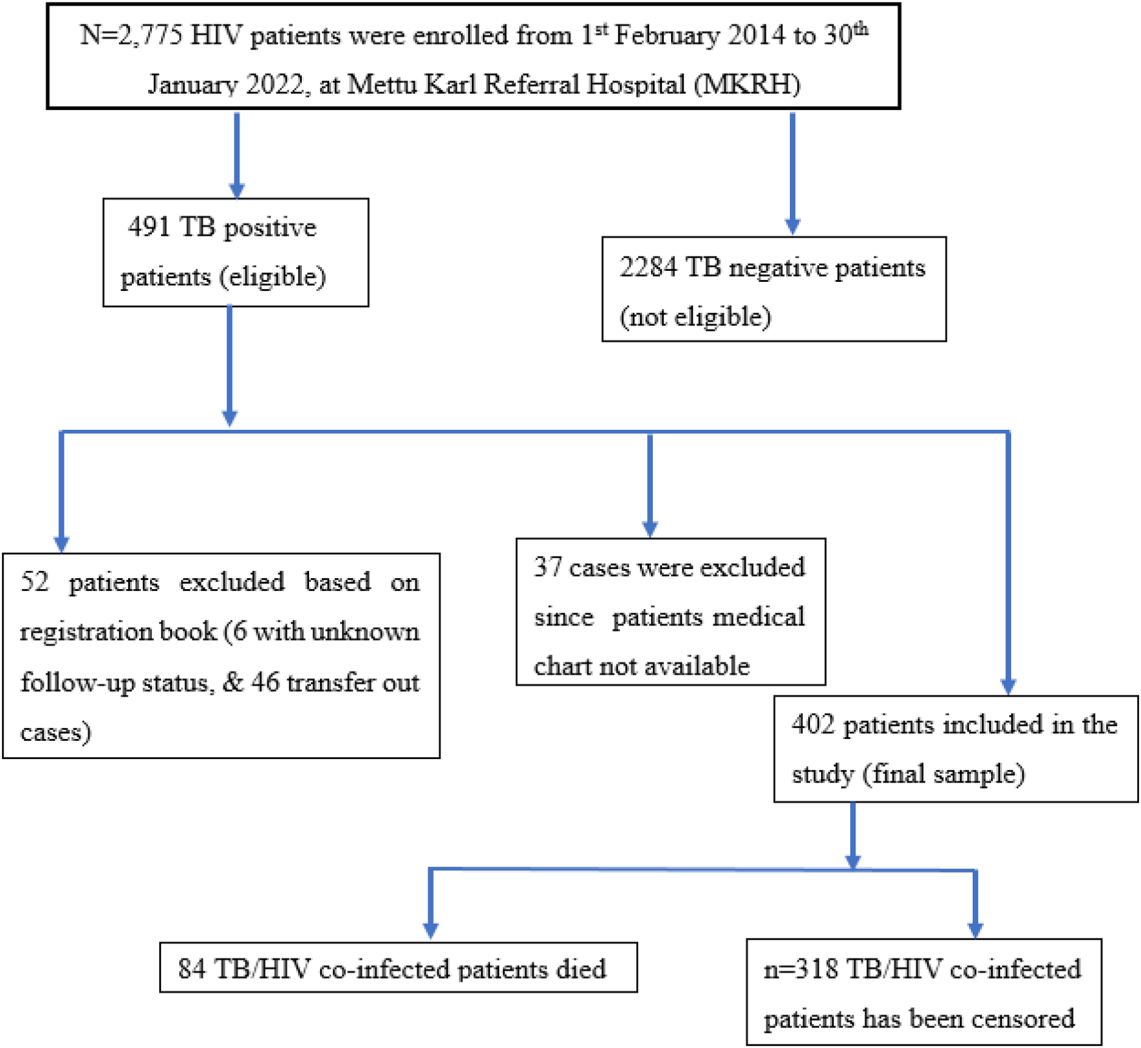
Overall enrollment of the adult patients co-infected with TB/HIV on ART at Mettu Karl Hospital, Ethiopia, 2014–2022 (n = 402).

All patients co-infected with TB and HIV who were registered and receiving follow-up care from 1 February 2014 to 30 January 2022 were included in the current study. During the study period, a total of 2775 individuals were enrolled in the ART clinic for HIV care. All HIV patients are routinely examined for tuberculosis, and 491 people were found to be TB/HIV coinfected during the same time period. Each patient in the cohort was retrospectively studied starting from the initiation of TB/HIV co-infection until death, loss to follow-up, or the end of the study period. Since it is known that stopping ART during TB therapy raises the chance of death by many times, those who did so were eliminated. Additionally, those who lacked complete disease documentation were excluded from the study. We provided a brief explanation in the diagram (Fig. 1). As a result, 89 were rejected due to the aforementioned exclusion criteria. As a result, 402 patients with both TB and HIV were included in the study. These patients' post-treatment survival times were calculated, and those who died from causes other than HIV/TB, were alive/not died at the end of the study, or whose data were unavailable after a certain period of time, were regarded as censored.

### Extraction of data and quality control

To extract data, the study employed a data extraction method that is adapted from standard registries of the national ART and anti-TB therapy. Sources of data included standard ART and anti-TB therapy registries, electronic formats, medical records of the patient (cards), and intake forms. Five nurses who are trained and worked in the hospital's ART and TB clinics participated in the data extraction process. An experienced supervisor had a follow-up on the extraction procedures, to ensure data quality. The checklists were continually reviewed for consistency and completeness by the researchers and the supervisor during the data extraction process. Data on baseline characteristics of sociodemographic, clinical, behavioral, and health services were taken from the sources.

### Variables in the study

#### Dependent variable

The dependent variable in the current study was the survival time to death of patients co-infected with TB and HIV from diagnosis to the end of the follow-up time.

#### Explanatory variables

The following were the predictor factors used in the current study: Patient demographics include sex, age of patients (in years), place of residence, marital status, religion, level of education, and employment status. Clinical parameters include the WHO clinical stage, a cluster of differentiation 4 (CD4 count/mm^3^), BMI, opportunistic infection, sickness other than tuberculosis, disclosure status, functional state, INH, and CPT use.

### Methods of data analysis

To report descriptive results, the current study used frequency, percentages, tables, and graphs. Further, the study used Kaplan–Meier to look at the difference between survival curves. Study subjects were followed until the end of the period, and at the end, subjects were dichotomized as either experiencing an event (death) or not (called censored). To identify predictors of survival time to death among TB and HIV co-infected patients Cox regression was utilized. Before proceeding with multivariable analysis univariable analysis was done for all covariates included in the study and covariates with a *p* value ≤ 25% and/or their clinical importance were identified as candidates for the final model. Covariates with a *p* value of less than 0.05 were considered significant in the final model.

### Ethical approval

Mettu University's College of Natural and Computational Sciences Research and Ethics Review Board granted ethical permission and human subject research approval for this study, with ethics approval number MeU/IRRB/016/22. This research was carried out per the Helsinki Declaration.

### Consent to participate

Mettu University Research Review Board waived the requirement for written informed consent of study participants, but data were kept anonymous and confidential.

## Results

### Baseline socio-demographic characteristics of study subjects

Of all study subjects included in the current study, 176(43.8%) were males, while more than half, 226(56.2%) were females of which 40(22.7%) and 44(19.5%) males and females have experienced death respectively. When we look at age of TB/HIV co-infected patients, a significant portion of 187(46.5%) were aged 25–34 years, of which 28(15.0%) died. A high percentage of death was recorded among patients those aged 45 years and above. More than half of the participants were rural dwellers, 218(54.2%), while 184(45.8%) were urban residents. Furthermore, in terms of religion, 103(25.6%), 101(25.1%), and 192(47.8%) were followers of Muslim, Protestant, Orthodox, and other religions, respectively. A large percentage of patients with 178(44.3%) were married. Out of 402 study individuals, 171(42.5%) had attended primary school followed by no formal education 127(31.6%). A large percentage of death was experienced among those who had no education (illiterate), with 35(27.6%). Regarding the employment status of patients, more than half, 229(57.0%) were full-time workers followed by 70(17.4%) patients who were not working due to different reasons like ill health and/or studying (Table 1).

**Table 1:** The baseline demographic characteristics of study subjects of TB/HIV co-infected adults on ART at Mettu Karl Referral Hospital, Ethiopia, 2014–2022 (n = 402).

Covariates	Categories	Total n (%)	Survival status	
			Censored	Died
			n (%)	n (%)
Sex	Male	176 (43.8)	136 (77.3)	40 (22.7)
	Female	226 (56.2)	182 (80.5)	44 (19.5)
Age	15–24	54 (13.4)	45 (83.3)	9 (16.7)
	25–34	187 (46.5)	159 (85.0)	28 (15.0)
	35–44	117 (29.1)	85 (72.6)	32 (27.4)
	> 45	44 (10.9)	29 (65.9)	15 (34.1)
Residence	Urban	184 (45.8)	123 (66.8)	61 (33.2)
	Rural	218 (54.2)	195 (89.4)	23 (10.6)
Religion	Muslim	103 (25.6)	78 (75.7)	25 (24.3)
	Protestant	101 (25.1)	73 (72.3)	28 (27.7)
	Orthodox	192 (47.8)	162 (84.4)	30 (15.6)
	Others	6 (1.5)	5 (83.3)	1 (16.7)
Marital	Never married	103 (25.6)	76 (73.8)	27 (26.2)
	Married	178 (44.3)	154 (86.5)	24 (13.5)
	Separated	56 (13.9)	46 (82.1)	10 (17.9)
	Divorced	41 (10.2)	28 (68.3)	13 (31.7)
	Widow	24 (6.0)	14 (58.3)	10 (41.7)
Education status	No education	127 (31.6)	92 (72.4)	35 (27.6)
	Primary	171 (42.5)	143 (83.6)	28 (16.4)
	Secondary	79 (19.7)	64 (81.0)	15 (19.0)
	Tertiary	25 (6.2)	19 (76.0)	6 (24.0)
Employment	Working full time	229 (57.0)	198 (86.5)	31 (13.5)
	Working part-time	62 (15.4)	47 (75.8)	15 (24.2)
	Not working/studying/due to ill health	70 (17.4)	43 (61.4)	27 (38.6)
	Unemployed	41 (10.2)	30 (73.2)	11 (26.8)

### Clinical features of TB and HIV co-infected adult patients

Our findings showed that nearly half of the participants, 192(47.8%) had WHO clinical stage III followed by WHO clinical stage IV, 75(18.7%). Of all study participants, patients with a CD4 count < 200 cells/mm^3^ made up 205(51.0%) of the total study participants, of which 47(56.0%). Out of 282(70.1%) cotrimoxazole preventive therapy (CPT) user participants, the proportion of death was 12.1%, while among non-users was 41.7%. Patients who were treated with Isoniazid or isonicotinic acid hydrazide (INH) were 126(31.3%). Almost half 203(50.5%) of study participants had normal weight, followed by underweight with 183(45.5%). Regarding the functional status of participants, 212(52.7%), 136(33.8%), and 54(13.4%) were working, ambulatory, and bedridden respectively. Around three-fourth, 293(72.9%) of participants disclosed TB treated while 109(27.1%) were not. A significant proportion, 218(54.2%) of the study subjects had opportunistic infections (one or more illnesses) other than TB, of which 52(61.9%) died (Table 2).

**Table 2:** Summaries of clinical features of TB and HIV co-infected adults on ART at Mettu Karl Referral Hospital, Ethiopia, 2014–2022 (n = 402).

Covariates	Categories	Total n (%)	Survival status	
			Censored	Died
			n (%)	n (%)
WHO	WHO stage I	45 (11.2)	41 (12.9)	4 (4.8)
	WHO stage II	90 (22.4)	74 (23.3)	16 (19.0)
	WHO stage III	192 (47.8)	154 (48.4)	38 (45.2)
	WHO stage IV	75 (18.7)	49 (15.4)	26 (31.0)
CD4	< 200 count/mm^3^	205 (51.0)	158 (49.7)	47 (56.0)
	≥ 200 count/mm^3^	197 (49.0)	160 (50.3)	37 (44.0)
CPT	No	120 (29.9)	70 (58.3)	50 (41.7)
	Yes	282 (70.1)	248 (87.9)	34 (12.1)
INH	No	276 (68.7)	203 (63.8)	73 (86.9)
	Yes	126 (31.3)	115 (36.2)	11 (13.1)
BMI	< 18.5	183 (45.5)	127 (39.9)	56 (66.7)
	18.5–25	203 (50.5)	176 (55.3)	27 (32.1)
	> 25	16 (4.0)	15 (4.7)	1 (1.2)
Function	Working	212 (52.7)	178 (56.0)	34 (40.5)
	Ambulatory	136 (33.8)	109 (34.3)	27 (32.1)
	Bedridden	54 (13.4)	31 (9.7)	23 (27.4)
Disclosure	No	109 (27.1)	80 (25.2)	29 (34.5)
	Yes	293 (72.9)	238 (74.8)	55 (65.5)
Opportunistic infection	No	184 (45.8)	152 (47.8)	32 (38.1)
	Yes	218 (54.2)	166 (52.2)	52 (61.9)
Total	402 (100)	318 (79.1)	84 (20.9)	

### Survival status of TB, and HIV co-infected adult patients

The study results revealed that out of 402 adult patients co-infected with TB and HIV who were followed for 96 months, 84(20.9%) patients died, and 318 (79.1%) were censored (Table 2). Twenty-nine (29/84, 34.5%) deaths occurred among co-infected patients in the first five-month period of starting treatment of anti-TB. The study participants were followed for a total of 6920 person-months. It is also revealed that adult patients with TB and HIV co-infections had an overall incidence rate of 12.1 per 1000 person-months with a 95% confidence interval of [9.77–14.98]. Survival rates were 91%, 88%, and 85% respectively, at 5, 15, and 25 months after initiation of TB treatment (Table 3).

**Table 3:** The overall life table of adult patients co-infected with TB, and HIV on ART follow-up at Mettu Karl Referral Hospital, Ethiopia, 2014–2022 (n = 402). *Source*: MKRH from 1st February 2014 to 30th January 2022, CPS: Cumulative Proportion Surviving at End of Interval.

Interval start time (months)	No. exposed to risk	No. of events	Proportion events	Proportion surviving	*CPS
0	390.50	29	.07	.93	.93
5	343.00	7	.02	.98	.91
10	318.00	13	.03	.97	.88
15	293.00	5	.02	.98	.85
20	274.50	3	.04	.96	.80
25	252.50	5	.02	.98	.78
30	235.00	4	.02	.98	.76
35	217.50	1	.00	1.00	.76
40	203.50	3	.01	.99	.75

### Overall Kaplan–Meier curve of adult patients co-infected with TB/HIV

**Figure 2:**
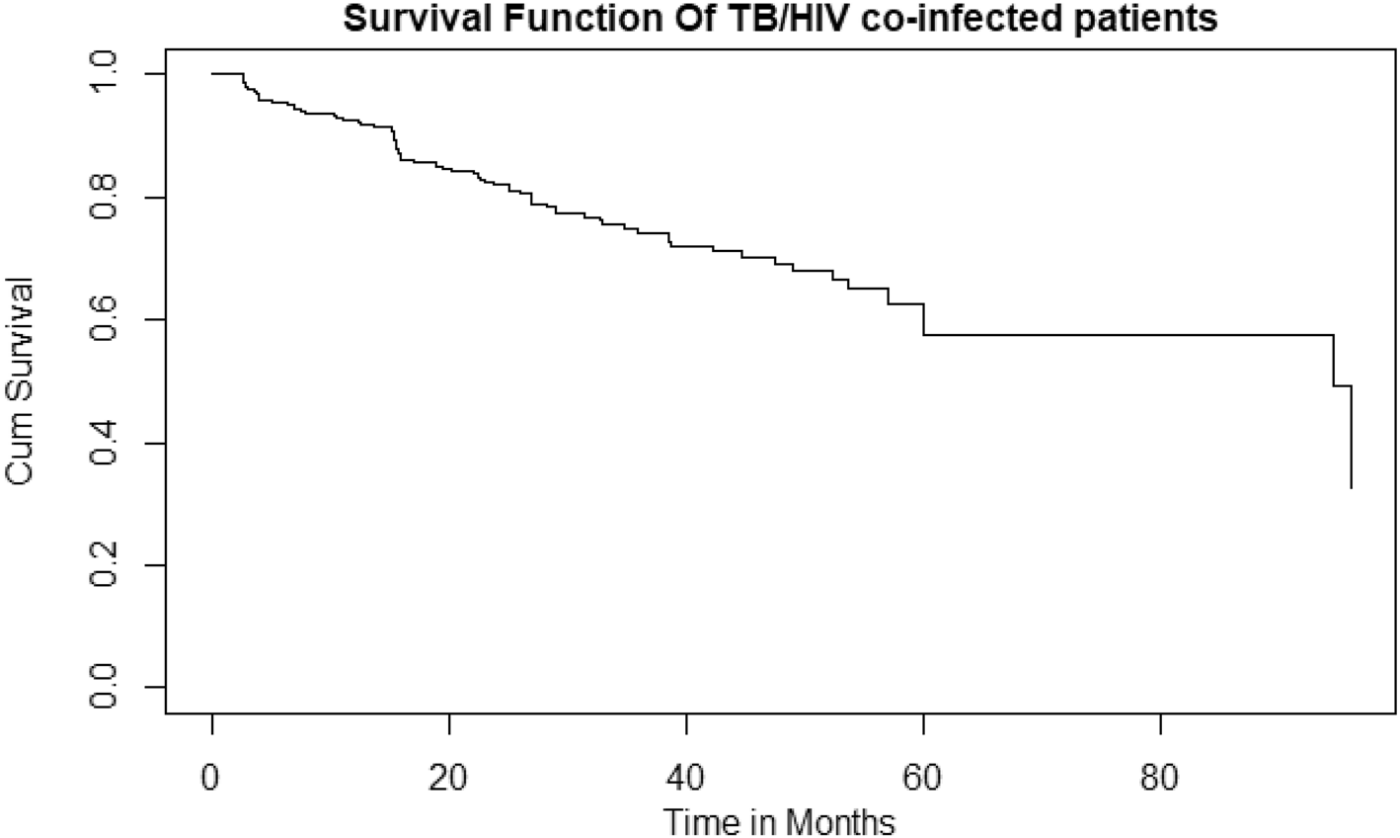
Overall Kaplan–Meier curve of survival proportion of TB/HIV co-infected adults on ART at Mettu Karl Referral Hospital, Ethiopia, 2014–2022 (n = 402).

The overall survival curve is continuously falling, implying that survival time is decreasing over time. According to the findings of the current study, adult patients who were co-infected with TB and HIV had a median survival period of 17.6 months (Fig. 2).

### Kaplan–Meier curves of some significant variables of TB, and HIV co-infected adult patients

**Figure 3:**
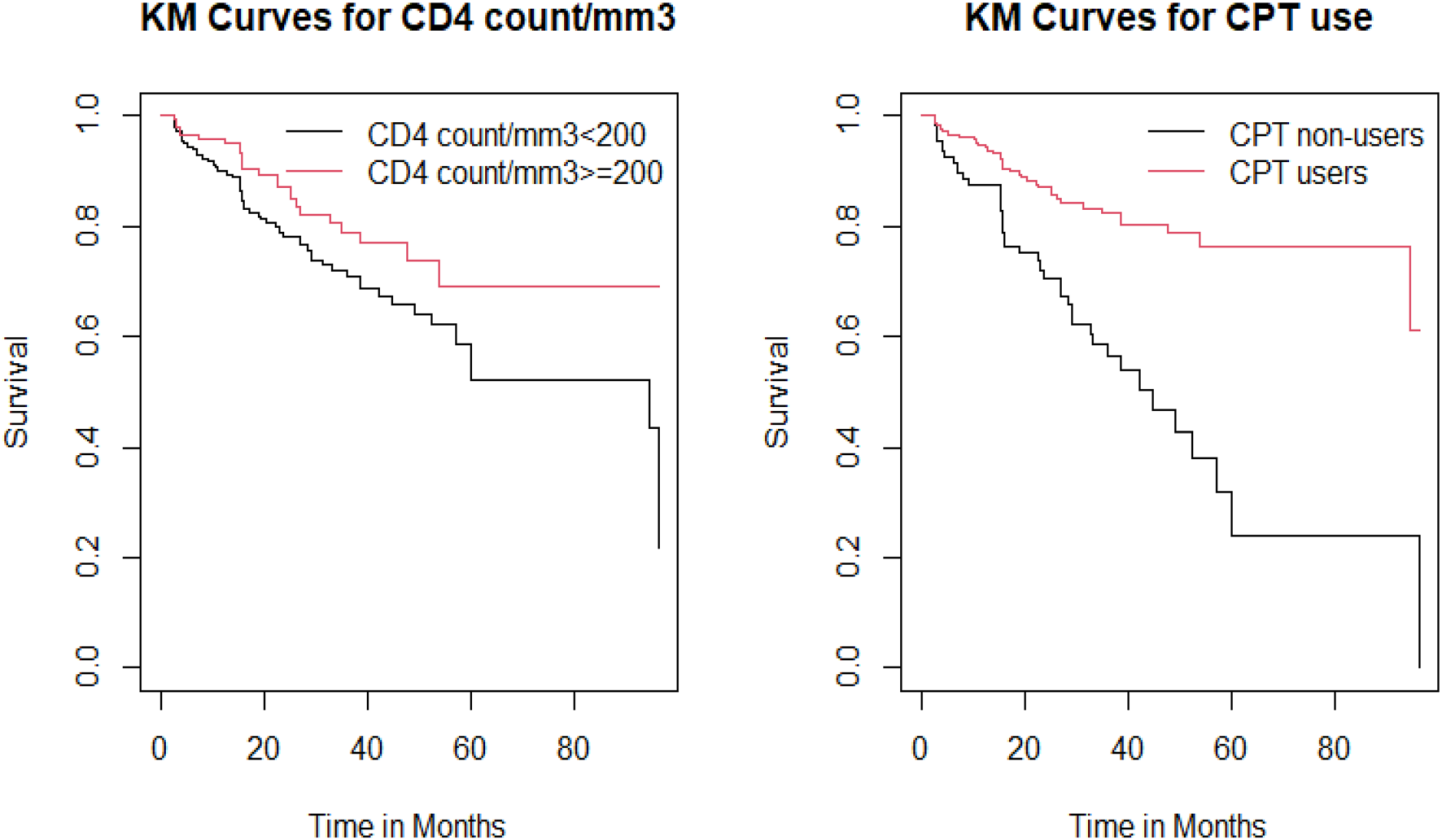
Survival curves for CD4 count/mm^3^, and CPT use of TB, and HIV co-infected adults who were on ART at Mettu Karl Referral Hospital, Ethiopia, 2014–2022 (n = 402).

Some of the significant covariates such as CD4 count/mm3 and Cotrimoxazole Prophylactic Therapy (CPT), were represented as Kaplan Meier curves (Fig. 3). We may use these graphs to see if the survival time of TB/HIV co-infected patients varies by subgroup. Accordingly, the survival curve of TB/HIV co-infected patients with CD4 count/mm^3^ ≥ 200 lies above those with CD4 count/mm^3^ < 200. It suggests that those with a CD4 count/mm^3^ of greater than 200 had a considerably prolonged survival time to death than those with a CD4 count/mm^3^ of less than 200. In addition, there appears to be a difference in survival time between those who utilize Cotrimoxazole Prophylactic Therapy (CPT) and those who do not. CPT users had a longer survival time than non-users, implying that they are relatively at a lower risk of mortality than CPT non-users.

### Predictors of mortality among HIV and TB co-infected patients

TB/HIV co-infected individuals aged above 45 had a 2.054(AHR = 2.054; 95%CI: 1.025–4.115) times higher probability of death than those aged 15–24. Patients who lived in urban areas were 0.422(AHR = 0.422; 95%CI: 0.282–0.633) times less likely to die than those who lived in rural areas. Patients who have a CD4 count ≥ 200 cells/mm^3^ (AHR = 0.719; 95% CI: 0.536–0.966) have a lower risk of dying than those who have a CD4 count < 200 cells/mm^3^.

The risk of death is 2.596 (AHR = 2.596, 95%CI: 1.175–5.734) and 1.344 (AHR = 1.344; 95%CI: 1.024–5.370) times higher for co-infected patients with WHO clinical stages IV and II, respectively than it is for patients with WHO clinical stage I. Bedridden patients had a mortality risk that was 2.188 (AHR = 2.188; 95% CI: 1.353–3.539) times higher than those who were working. Moreover, the current study discovered that patients with TB/HIV co-infection who were receiving INH had a 0.310 (AHR = 0.310; 95%CI: 0.168–0.574) times lower mortality rate than those who were not receiving INH (Table 4).

**Table 4:** Results of Multivariable Cox-regression analysis for predictors of survival time to death among TB/HIV co-infected adult patients on ART at Mettu Karl Referral Hospital, Ethiopia, 2014–2022 (n = 402). *Source*: MKRH from 1st February 2014 to 30th January 2022, AHR: Adjusted Hazard Ratio.

Covariates	Categories	Coef	AHR	Se(coef)	*p* value	$95\mathrm{\%CI\, for\, A}HR$
Gender of patients (Ref: female)	Male	− 0.218	0.804	0.171	0.509	[0.575, 1.124]
Age of patients (in years) (Ref: 15–24)	25–34	0.178	1.194	0.317	0.576	[0.642, 2.221]
	35–44	0.213	1.237	0.328	0.517	[0.650, 2.354]
	≥ 45	0.719	2.054	0.355	0.042	[1.025, 4.115]
Residence (Ref: rural)	Urban	− 0.863	0.422	0.207	< 0.001	[0.282, 0.633]
Education (Ref: No education)	Primary	− 0.234	0.791	0.228	0.303	[0.506, 1.236]
	Secondary	− 0.236	0.790	0.261	0.367	[0.473, 1.318]
	Tertiary	− 0.179	0.835	0.425	0.672	[0.363, 1.922]
CD4 count/mm3 (Ref: < 200)	≥ 200	− 0.329	0.719	0.190	0.008	[0.536, 0.966]
WHO clinical stages (Ref: I)	II	0.852	1.344	0.423	0.044	[1.024, 5.370]
	III	0.518	1.679	0.386	0.179	[0.788, 3.577]
	IV	0.954	2.596	0.404	0.018	[1.175, 5.734]
Opportunistic infection (Ref: No)	Yes	− 0.088	0.916	0.035	0.653	[0.855, 0.981]
Functional status (Ref: working)	Ambulatory	0.252	1.286	0.226	0.055	[0.826, 2.01]
	Bedridden	0.783	2.188	0.245	0.001	[1.353, 3.539]
INH (Ref: No)	Yes	− 1.169	0.310	0.314	< 0.001	[0.168, 0.574]
CPT use (Ref: No)	Yes	− 0.627	0.534	0.198	0.0185	[0.363, 0.787]
Status of disclosure (Ref: No)	Yes	0.041	1.042	0.192	0.832	[0.715, 1.518]

Coef: coefficients, AHR: Adjusted hazard ratio, Se(coef): standard error of coefficients, CI: 95% confidence interval for AHR(Adjusted Hazard Ratio).

## Discussion

The purpose of this study was to look at an eight-year retrospective cohort of Tuberculosis and HIV co-infected patients on ART to learn more about survival rates and predictors of mortality in the hospital setting at Mettu Karl Referral Hospital. To estimate and compare survival times, the Kaplan Meier survival curve and log-rank tests were utilized. For each explanatory variable, a bivariate Cox-proportional hazards regression model was fitted, and a multivariable Cox model was utilized to find predictors of mortality among TB/HIV co-infected subjects. To determine the strength of the link, the adjusted Hazard Ratio with its 95 percent confidence interval was employed, and the *p* value of 0.05 was considered statistically significant. The assumption of proportional hazard was examined and found to be satisfactory.

In this retrospective analysis, the death rate was significantly high, with 20.9% of TB, and HIV co-infected patients dying during the follow-up period. This is in agreement with prior studies in various parts of the world. For instance; death rates reported from Jimma University Teaching Hospital were 20.2%^18^, Bahir Dar, Northwest Ethiopia 18.0%^6^, a multi-center study from Southwest Ethiopia 21.8%^24^, and from Mekelle, Northern Ethiopia 23.0%^3^, Malawi 20%^25^. However, it is significantly higher than the reported study conducted in the southwest part of Ethiopia (6.4%)^26^, Somali Region, Eastern Ethiopia, 11.1%^27^, Northwest Ethiopia (4.2%)^28^, South Omo, Ethiopia, 10%^29^, Harar town 7.7%^1^, and Maynmar 13.7%^30^. But, when compared to this result, a study in Debre Markos reported higher findings (43%)^31^. These disparities might be attributed to differences in the time follow-up, research locations, and availability facility.

A significant proportion of the deaths, 29 (34.5%), took place within the first five months of starting anti-TB medication. This is more than twice as high as the South African findings^32^, however, it is lower than the results from Mizan-Tepi University teaching hospital^4^. This suggests that pharmacological interactions and overlapping toxicities of anti-TB and ART treatments, immunological reconstitution inflammatory syndrome, and other opportunistic infections, all of which have been linked to early death among TB/HIV co-infected patients, should be managed appropriately. In addition, our findings showed that the median time until death was found to be 17.6 months, which is longer than the previous study's finding which was 10 months reported from the Mizan-Tepi university teaching hospital^4^. Another multicenter study from southwest Ethiopia reported that the median time of death was 15.6 months, which is comparable to the results found in this study^24^. The overall incidence rate was 12.1 per 1000 person months. The incidence rates per 1000 person-year were 3.0 and 5.4, respectively, in studies done at Dilla University Hospital^33^ and seven Ethiopian university teaching hospitals^34^. This indicated that the incidence rate was higher in the current study, which may have been due to differences in the study's design and the hospitals' facility levels.

In our investigation, the age of patients was revealed to be a major factor associated with the death of TB/HIV co-infected people. TB/HIV co-infected individuals aged above 45 had a 2.054 higher probability of death than those aged 15–24. This could be attributed to patients' declining immunity as they become older. A study conducted in Southwest Ethiopia^18^, and Brazil^21^, found that study participant aged older than 65 had a nearly threefold greater risk of death when compared to those younger patients. According to an Indian study, patients aged 41–60 years and those above 60 years were 7.8 and 21.34 times more likely to die earlier than patients aged less than 20 years^35^. A previous study^2^ discovered that the death rate of TB and HIV co-infection increased by 4% for every year of age added. This may be due to the fact that elderly people are more likely to be diagnosed with HIV and/or tuberculosis later in life. It is well recognized that diagnosis at a later stage contributes to poor prognosis and mortality due to immunological weakness caused by rapid progression to AIDS and extra-pulmonary tuberculosis^36–39^.

This study indicated that among HIV-positive patients who are on ART, residency was revealed to be a major factor in death. Patients who lived in urban areas were 0.422 times less likely to die than those who lived in rural areas. This is supported by findings of previous studies^24,40^. This could be related to rural people's lack of access to TB services to ensure quick diagnosis and treatment in resource-constrained locations.

As observed in this analysis, the CD4 cell count has a significant association with survival rates, and studies argued that a low CD4 cell count was associated with a higher risk of mortality^20,41–43^. TB and HIV infection have a bidirectional and synergistic effect on the survival time of TB/HIV co-infected patients. HIV captures CD4 cells activated by latent or active TB, and HIV depletes CD4 cells, hastening the progression of latent TB to the form of active TB illness. Similar to this report, patients who have a CD4 count ≥ 200 cells/mm^3^ have a lower risk of dying than those who have a CD4 count < 200 cells/mm^3^. According to a study^22^ done in Barcelona, Spain, patients who had CD4 cell counts under 200/mm^3^ had lower chances of surviving^22,44^. This is due to the fact that a low CD4 count makes it challenging for the body to combat additional infections, making even a straightforward infection like a cold significantly worse. The body's inability to react to fresh infections is the cause of this.

The current investigation found a significant statistical relationship between the WHO clinical stage with the risk of death from TB-HIV co-infection. The risk of death is 2.596 and 1.344 times higher for co-infected patients with WHO clinical stages IV and II, respectively than it is for patients with WHO clinical stage I. This is consistent with earlier studies that found that advanced WHO clinical stages increased death among TB patients who live with HIV^1,45^. To a report of a study done at the University of Gondar Hospital, TB-positive people who live with HIV and who had a WHO clinical stage of IV were an 8.6 times higher probability of death as compared to those with stage III^46^. This may be due to the increased risk of opportunistic infections in people with advanced WHO clinical stages.

In the literature like^20,43,47,48^, it is found that the functional status of bedridden people with TB and HIV co-infection had a significantly higher death risk compared to those who were at working status. Similarly, previous research conducted at Jimma, Southwest Ethiopia found that patients who were bedridden in functional status had a threefold greater risk of dying than those who were at working status^18^. Current study findings were in line with an investigation carried out in Bahir Dar, Ethiopia^6^, which found that patients who were bedridden had a mortality risk that was roughly four times higher than those who were working. These results are not surprising because bedridden patients have a worse prognosis for health because of a relentless immunity-lowering cycle, which can result in patients contracting opportunistic infections and dying^49^. Therefore, it makes sense to support regular and necessary opportunistic infection and other illness screening to deploy prompt and effective management techniques. and decrease avoidable deaths.

Additionally, the current study discovered that patients with TB/HIV co-infection who were receiving INH had a 0.310 times lower mortality rate than those who were not receiving INH. Individuals with HIV and drug-susceptible TB should get standard anti-TB therapy, which includes 2 months of isoniazid (INH), rifampin, pyrazinamide, and ethambutol (intensive phase), followed by 4 months of isoniazid (INH) and rifampin (continuation phase)^50^. Studies from various parts of the world reported CPT as a significant predictor of mortality among patients co-infected with TB and HIV. For instance, according to research from South India and Sub-Saharan Africa, not taking CPT significantly increased mortality^51,52^. Another study from Harar, Ethiopia reported that those who did not take co-trimoxazole prophylaxis (CPT) had a greater risk of having their TB treatment fail. Our research supported these reports by demonstrating a relationship between taking CPT during TB treatment and a decreased risk of death in TB/HIV co-infected people^1^. The risk of death was 0.534 times lower for patients who received CPT compared to those who did not. This finding is consistent with a study^24^, conducted in southwest Ethiopia. Cotrimoxazole preventive therapy significantly increased patient survival, according to a study carried out in Tehran, Iran. In their findings, patients who did not receive preventive therapy had a 3.68 times greater risk of passing away than those who did^53^. The greater risk of mortality among people who are CPT nonusers may be due to malaria and serious bacterial infections, both of which can be effectively prevented both from happening by co-trimoxazole preventative treatment^14,54,55^. In tuberculosis patients with HIV, co-trimoxazole preventative treatment (CPT) lowers morbidity and death.

## Limitations, and strengths of the study

The retrospective nature of the study might lead to selection bias. Also, some socio-economic parameters like situations of living, social support, and distance of health centers were not documented, but they might be factors for survival based on literature. Further variables on this issue, a larger sample, and multicenter studies are recommended for studies to give a better conclusion.

## Conclusion

In this retrospective study, 20.9 percent of the patients on ART died during the follow-up period, which is still high. The overall incidence rate in the current study was 12.1 per 1000 person months. It is also shown that high mortality of TB and HIV co-infected patients occurred in the earlier months of initiation of treatment. To reduce TB-related mortality, targeted therapies that can keep patients free of TB in the early phases of treatment are needed. Patients' survival can be improved if those with low CD4 counts, those in advanced WHO stages, and those with poor functional levels are continuously followed and if CPT is begun immediately with encouraging HIV status disclosure.

## Data Availability

All data generated and used in the current study are available from the corresponding author upon reasonable request.

## Abbreviations

AHR: Adjusted hazard ratio
AIDS: Acquired immunodeficiency syndrome
ART: Antiretroviral therapy
BMI: Body mass index
CD4: Cluster of differentiation
CPT: Cotrimoxazole prophylactic therapy
HIV: Human immunodeficiency virus
INH: Isonicotinic acid hydrazide
MKRH: Mettu Karl Referral Hospital
mm^3^: Millimeter cube
OIs: Opportunistic infections
TB: Tuberculosis
WHO: World Health Organization
SSA: Sub-Saharan Africa

## References

[1] Tola A, Mishore KM, Ayele Y, Mekuria AN, Legese N (2019). Treatment outcome of tuberculosis and associated factors among TB-HIV Co-infected patients at public hospitals of Harar town, Eastern Ethiopia. A five-year retrospective study. BMC Public Health.

[2] Mugusi FM, Mehta S, Villamor E, Urassa W, Saathoff E, Bosch RJ, Fawzi WW (2009). Factors associated with mortality in HIV-infected and uninfected patients with pulmonary tuberculosis. BMC Public Health.

[3] Gezae KE, Abebe HT, Gebretsadik LG, Gebremeskel AK (2020). Predictors of time to death among TB/HIV co-infected adults on ART at two governmental hospitals in Mekelle, Ethiopia, 2009–2016: A retrospective cohort study. Ann. Infect. Dis. Epidemiol..

[4] Wondimu W, Dube L, Kabeta T (2020). Factors affecting survival rates among adult TB/hiv co-infected patients in Mizan Tepi University teaching hospital, South West Ethiopia. HIV/AIDS (Auckland, NZ).

[5] Kwan CK, Ernst JD (2011). HIV and tuberculosis: A deadly human syndemic. Clin. Microbiol. Rev..

[6] Sileshi B, Deyessa N, Girma B, Melese M, Suarez P (2013). Predictors of mortality among TB-HIV Co-infected patients being treated for tuberculosis in Northwest Ethiopia: A retrospective cohort study. BMC Infect. Dis..

[7] Deribew A, Tesfaye M, Hailmichael Y, Apers L, Abebe G, Duchateau L, Colebunders R (2010). Common mental disorders in TB/HIV co-infected patients in Ethiopia. BMC Infect. Dis..

[8] Deribew A, HaileMichael Y, Tesfaye M, Desalegn D, Wogi A, Daba S (2010). The synergy between TB and HIV co-infection on perceived stigma in Ethiopia. BMC Res. Notes..

[9] J.U.N.P. on HIV/AIDS, Global AIDS Monitoring 2017: Indicators for monitoring the 2016 United Nations Political Declaration on HIV and AIDS. Geneva: Joint United Nations Programme on HIV, AIDS (2016).

[10] W.H. Organization, Global tuberculosis report 2013, World Health Organization (2013).

[11] Kassu A, Mengistu G, Ayele B, Diro E, Mekonnen F, Ketema D, Moges F, Mesfin T, Getachew A, Ergicho B (2007). Coinfection and clinical manifestations of tuberculosis in human immunodeficiency virus-infected and-uninfected adults at a teaching hospital, northwest Ethiopia. J. Microbiol. Immunol. Infect..

[12] Demissie, M., Lindtjørn, B. & Tegbaru, B. Human immunodeficiency virus (HIV) infection in tuberculosis patients in Addis Ababa, Ethiop. *J. Heal. Dev.* **14** (2000).

[13] Jacobs GB, Ikomey GM (2016). Biomedical research and capacity building: Bilateral collaboration between research institutes in South Africa and Cameroon. SAMJ S. Afr. Med. J..

[14] W.H. Organization, Consolidated guidelines on the use of antiretroviral drugs for treating and preventing HIV infection: Recommendations for a public health approach, World Health Organization (2016). 27466667

[15] Teklu AM, Nega A, Mamuye AT, Sitotaw Y, Kassa D, Mesfin G, Belayihun B, Medhin G, Yirdaw K (2017). Factors associated with mortality of TB/HIV co-infected patients in Ethiopia, Ethiop. J. Health Sci..

[16] Mitku AA, Dessie ZG, Muluneh EK, Workie DL (2016). Prevalence and associated factors of TB/HIV co-infection among HIV Infected patients in Amhara region, Ethiopia. Afr. Health Sci..

[17] Beyene Y, Geresu B, Mulu A (2016). Mortality among tuberculosis patients under DOTS programme: A historical cohort study. BMC Public Health.

[18] Gesesew H, Tsehayneh B, Massa D, Gebremedhin A, Kahsay H, Mwanri L (2016). Predictors of mortality in a cohort of tuberculosis/HIV co-infected patients in Southwest Ethiopia. Infect. Dis. Poverty.

[19] Shaweno D, Worku A (2012). Tuberculosis treatment survival of HIV positive TB patients on directly observed treatment short-course in Southern Ethiopia: A retrospective cohort study. BMC Res. Notes.

[20] Refera H, Wencheko E (2013). Survival of HIV-TB co-infected adult patients under ART in Ambo Referral Hospital, Ethiopia. Ethiop. J. Heal. Dev..

[21] Domingos MP, Caiaffa WT, Colosimo EA (2008). Mortality, TB/HIV co-infection, and treatment dropout: Predictors of tuberculosis prognosis in Recife, Pernambuco State, Brazil. Cad. Saude Publ..

[22] Català L, Orcau A, García de Olalla P, Millet JP, Rodríguez-Mondragón A, Caylà JA, Group T-HW (2011). Survival of a large cohort of HIV-infected tuberculosis patients in the era of highly active antiretroviral treatment. Int. J. Tuberc. Lung Dis..

[23] Mekonnen D, Derbie A, Desalegn E (2015). TB/HIV co-infections and associated factors among patients on directly observed treatment short course in Northeastern Ethiopia: A 4 years retrospective study. BMC Res. Notes.

[24] Lelisho, M. E., Teshale, B. M., Tareke, S. A., Hassen, S. S., Andargie, S. A., Merera, A. M. & Awoke, S. Modeling survival time to death among TB and HIV co-infected adult patients: An institution-based retrospective cohort study. *J. Racial Ethn. Heal. Disparities* 1–13 (2022). 10.1007/s40615-022-01348-w 35697902

[25] Zachariah R, Fitzgerald M, Massaquoi M, Acabu A, Chilomo D, Salaniponi FML, Harries AD (2007). Does antiretroviral treatment reduce case fatality among HIV-positive patients with tuberculosis in Malawi?. Int. J. Tuberc. Lung Dis..

[26] Gesesew HA, Ward P, Woldemichael K, Mwanri L (2018). Early mortality among children and adults in antiretroviral therapy programs in Southwest Ethiopia, 2003–15. PLoS ONE.

[27] Damtew, B., Mengistie, B. & Alemayehu, T. Survival and determinants of mortality in adult HIV/Aids patients initiating antiretroviral therapy in Somali Region, Eastern Ethiopia. *Pan Afr. Med. J.* **22** (2015). 10.11604/pamj.2015.22.138.4352 PMC4742016 26889319

[28] Assemie MA, Muchie KF, Ayele TA (2018). Incidence and predictors of loss to follow up among HIV-infected adults at Pawi General Hospital, northwest Ethiopia: Competing risk regression model. BMC Res. Notes.

[29] Tachbele, E. & Ameni, G. Survival and predictors of mortality among human immunodeficiency virus patients on anti-retroviral treatment at Jinka Hospital, South Omo, Ethiopia: A six years retrospective cohort study. *Epidemiol. Health* **38** (2016). 10.4178/epih.e2016049 PMC5309728 27820957

[30] Aung ZZ, Saw YM, Saw TN, Oo N, Aye HNN, Aung S, Oo HN, Cho SM, Khaing M, Kariya T (2019). Survival rate and mortality risk factors among TB–HIV co-infected patients at an HIV-specialist hospital in Myanmar: A 12-year retrospective follow-up study. Int. J. Infect. Dis..

[31] Tadele A, Shumey A, Hiruy N (2014). Survival and predictors of mortality among adult patients on highly active antiretroviral therapy at debre-markos referral hospital, North West Ethiopia; A retrospective cohort study. J. AIDS Clin. Res..

[32] Camara A, Sow MS, Touré A, Diallo OH, Kaba I, Bah B, Diallo TH, Diallo MS, Guilavogui T, Sow OY (2017). L’issue du traitement, la survie et ses facteurs de risque chez les nouveaux tuberculeux co-infectés par le VIH pendant l’épidémie d’Ebola à Conakry. Rev. Epidemiol. Sante Publique.

[33] Hailemariam S, Tenkolu G, Tadese H, Vata PK (2016). Determinants of survival in HIV patients: A retrospective study of Dilla University Hospital HIV cohort. Int. J. Virol. AIDS.

[34] Haileamlak A, Hagos T, Abebe W, Abraham L, Asefa H, Teklu AM (2017). Predictors of hospitalization among children on ART in Ethiopia: A Cohort study, Ethiop. J. Health Sci..

[35] Pardeshi, G. Survival analysis and risk factors for death in tuberculosis patients on directly observed treatment-short course (2009). 19584488

[36] Mojumdar K, Vajpayee M, Chauhan NK, Mendiratta S (2010). Late presenters to HIV care and treatment, identification of associated risk factors in HIV-1 infected Indian population. BMC Public Health.

[37] Xu X, Liu JH, Cao SY, Zhao Y, Dong XX, Liang Y, Lu ZX (2013). Delays in care seeking, diagnosis and treatment among pulmonary tuberculosis patients in Shenzhen, China. Int. J. Tuberc. Lung Dis..

[38] Raizada N, Chauhan LS, Babu BS, Thakur R, Khera A, Wares DF, Sahu S, Bachani D, Rewari BB, Dewan PK (2009). Linking HIV-infected TB patients to cotrimoxazole prophylaxis and antiretroviral treatment in India. PLoS ONE.

[39] Tarekegn, S. The effect of HAART on incidence of tuberculosis among HIV infected patients in Hawassa University Referral Hospital, Addis Ababa Addis Abba Addis Abba Univ. (2011).

[40] Nguyen DT, Jenkins HE, Graviss EA (2018). Prognostic score to predict mortality during TB treatment in TB/HIV co-infected patients. PLoS ONE.

[41] Mageda, K., Leyna, G. H. & Mmbaga, E. J. High initial HIV/AIDS-Related mortality and-its predictors among patients on antiretroviral therapy in the kagera region of Tanzania: A five-year retrospective cohort study. *AIDS Res. Treat.* **2012** (2012). 10.1155/2012/843598 PMC3437609 22973505

[42] Nansera D, Bajunirwe F, Elyanu P, Asiimwe C, Amanyire G, Graziano FM (2012). Mortality and loss to follow-up among tuberculosis and HIV co-infected patients in rural southwestern Uganda. Int. J. Tuberc. Lung Dis..

[43] Gezie LD (2016). Predictors of CD4 count over time among HIV patients initiated ART in Felege Hiwot Referral Hospital, northwest Ethiopia: Multilevel analysis. BMC Res. Notes.

[44] Maruza M, Albuquerque M, Braga MC, Barbosa MTS, Byington R, Coimbra I, Moura LV, Batista JDL, Diniz GTN, Miranda-Filho DB (2012). Survival of HIV-infected patients after starting tuberculosis treatment: A prospective cohort study. Int. J. Tuberc. Lung Dis..

[45] Iliyasu, Z. & Babashani, M. Prevalence and predictors of tuberculosis coinfection among HIV-seropositive patients attending the Aminu Kano Teaching Hospital, Northern Nigeria. *J. Epidemiol.* 903030070 (2009). 10.2188/jea.JE20080026 PMC3924118 19265273

[46] Wondimeneh Y, Muluye D, Belyhun Y (2012). Prevalence of pulmonary tuberculosis and immunological profile of HIV co-infected patients in Northwest Ethiopia. BMC Res. Notes.

[47] Temesgen A, Gurmesa A, Getchew Y (2018). Joint modeling of longitudinal CD4 count and time-to-death of HIV/TB co-infected patients: A case of Jimma University Specialized Hospital. Ann. Data Sci..

[48] Sinshaw Y, Alemu S, Fekadu A, Gizachew M (2017). Successful TB treatment outcome and its associated factors among TB/HIV co-infected patients attending Gondar University Referral Hospital, Northwest Ethiopia: an institution based cross-sectional study. BMC Infect. Dis..

[49] Alene KA, Nega A, Taye BW (2013). Incidence and predictors of tuberculosis among adult people living with human immunodeficiency virus at the University of Gondar Referral Hospital, Northwest Ethiopia. BMC Infect. Dis..

[50] Rojo P, Carpenter D, Venter F, Turkova A, Penazzato M (2020). The HIV drug optimization agenda: promoting standards for earlier investigation and approvals of antiretroviral drugs for use in adolescents living with HIV. J. Int. AIDS Soc..

[51] Harries, A. D., Zachariah, R. & Lawn, S. D. Providing HIV care for co-infected tuberculosis patients: A perspective from sub-Saharan Africa [State of the art series. Tuberculosis. Edited by ID Rusen. Number 3 in the series]. *Int. J. Tuberc. Lung Dis.* **13**, 6–16 (2009) . 19105873

[52] Vijay S, Kumar P, Chauhan LS, Narayan Rao SV, Vaidyanathan P (2011). Treatment outcome and mortality at one and half year follow-up of HIV infected TB patients under TB control programme in a district of South India. PLoS One.

[53] Roshanaei G, Ghannad MS, Poorolajal J, Mohraz M, Molaeipoor L (2017). Survival rates among co-infected patients with human immunodeficiency virus/tuberculosis in Tehran, Iran. Iran. J. Public Health..

[54] Dyer, O. Covid-19: Many poor countries will see almost no vaccine next year, aid groups warn. *BMJ Br. Med. J.* **371** (2020). 10.1136/bmj.m4809 33310819

[55] Hasse B, Walker AS, Fehr J, Furrer H, Hoffmann M, Battegay M, Calmy A, Fellay J, Di Benedetto C, Weber R (2014). Co-trimoxazole prophylaxis is associated with reduced risk of incident tuberculosis in participants in the Swiss HIV Cohort Study. Antimicrob. Agents Chemother..

